# STR-typing of ancient skeletal remains: which multiplex-PCR kit is the best?

**DOI:** 10.3325/cmj.2012.53.416

**Published:** 2012-10

**Authors:** Melanie Harder, Rebecca Renneberg, Patrick Meyer, Ben Krause-Kyora, Nicole von Wurmb-Schwark

**Affiliations:** 1 Graduate School “Human Development in Landscapes”, Christian-Albrechts-University, Kiel, Germany; 2 Institute of Legal Medicine, University Hospital of Schleswig-Holstein, Kiel, Germany,; 3 State criminal investigation department of Lower Saxony, Kiel, Germany; 4 Institute of Clinical Molecular Biology, Christian-Albrechts-University, Kiel, Kiel, Germany; * The first two authors contributed equally to this work.

## Abstract

**Aim:**

To comparatively test nine commercially available short tandem repeat (STR)-multiplex kits (PowerPlex 16, 16HS, ES, ESI17, ESX17, S5 [all Promega]; AmpFiSTR Identifiler, NGM and SEfiler [all Applied Biosystems]) for their efficiency and applicability to analyze ancient and thus highly degraded DNA samples.

**Methods:**

Fifteen human skeletal remains from the late medieval age were obtained and analyzed using the nine polymerase chain reaction assays with slightly modified protocols. Data were systematically compared to find the most meaningful and sensitive assay.

**Results:**

The ESI, ESX, and NGM kits showed the best overall results regarding amplification success, detection rate, identification of heterozygous alleles, sex determination, and reproducibility of the obtained data.

**Conclusion:**

Since application of these three kits enables the employment of different primer sequences for all the investigated amplicons, a combined application is recommended for best possible and – most importantly – reliable genetic analysis of ancient skeletal material or otherwise highly degraded samples, eg, from forensic cases.

Genetic analysis of skeletal remains in many cases presents a tremendous challenge for forensic genetics, as well as for ancient DNA researchers (1,2). In forensic case-work, DNA-analysis has repeatedly been shown to be the only way of getting some information from bodies when they are highly decomposed leaving no morphological chance for identification or when no data for dental comparisons are available. The analysis of short tandem repeats (STR) is currently the most common method and many studies have dealt with the application of STRs to investigate problematic, highly degraded samples (3,4). The same approach is necessary for investigating skeletal remains from mass graves (5-7). For classical ancient studies, ancient DNA (aDNA) analysis is the only method available to gain any information on eg, kinship or population genetics (8,9).

Usually, satisfying genetic profiles can be obtained when the DNA samples are well preserved, whereas in cases of poor DNA conditions only partial profiles can be detected (10). There are many different post-mortem processes and environmental factors that can lead to degradation of biological samples. Thus, many skeletal remains show no or minimal amounts of nuclear DNA that is usually highly degraded and contains additionally very often polymerase chain reactions (PCR), inhibitors (11,12). This might hamper the genetic analysis and can increase the risk of contaminations with modern DNA since minimal ancient DNA amounts can easily be overwhelmed by foreign DNA (13). In consequence, there is a great need for finding improved assays or experimental set ups and working in absolutely pure laboratory-conditions (12,14).

Until now, the methods for the application on low copy number DNA (LCN) and minimal traces have been constantly adapted, improved, and optimized (15). For example, the focus has been placed on mini-STRs with amplicon sizes between 70-280 bp, based on the principle that smaller fragments can often still be amplified when DNA is already highly degraded (16-18). Nowadays, a wide range of commercially available STR-kits and self-made PCRs, especially developed for highly degraded samples is available (19-22). However, there is a considerable difference in efficiency, costs, and the processing time for each assay. Additionally, not every PCR kit is comparably suited for STR analysis from old bone or tooth material.

We applied nine of the currently most common commercially available PCR multiplex kits from different companies: PowerPlex16, 16HS, ES, ESI17, ESX17, S5 (Promega, Mannheim, Germany); AmpFiSTR Identifiler, NGM, and SEfiler (Applied Biosystems, Darmstadt, Germany) to the analysis of human skeletal remains from the late medieval age to find the best testing system regarding informational content, efforts, and expenses.

## Material and methods

### DNA material

The samples from 15 adult individuals (7 female) were used. Most samples (n = 10) came from the site Diepensee (Germany), which was excavated in 2004 during the Berlin Airport expansion. Nearby the old airport, a small village was discovered that could be dated to the 12th-13th century, ie, to the late Middle Ages. Besides a church and some pottery remains, a huge graveyard with approximately 400 skeletal remains was found (23).

Besides the individuals from the site Diepensee, one skeleton was excavated in Eldena and other four in Horno. These five individuals belong to the 12th-15th century. Two teeth per individual (sample a + b) were obtained for genetic investigations leading to two independent extraction samples. Thus, from every sample two different extracts were subjected to the 9 different PCRs enabling a real comparison of typing success.

### Prevention of contamination

To minimize the risk of contamination stringent precautions were followed. All steps from the extraction to the PCR were performed in separate rooms in highly clean and DNA-free conditions as possible. Surfaces and laboratory equipment were thoroughly cleaned with 0.2% DanKlorix (Colgate-Palmolive, Hamburg, Germany), a very useful chemical to remove unwanted DNA (24). Afterwards the equipment was additionally exposed to UV-irradiation (λ = 254 nm) for 30-minute prior to every use. Only commercially-certified DNA/RNA-free consumables were used and standard contamination prevention protocols were followed, including the use of disposable overalls, masks, shoe covers, and gloves (8,25,26). Post-PCR steps were carried out separately from all pre-PCR steps following the “one-way traffic” (from the pre-PCR to the post-PCR procedures) (27). Only staff with a known DNA profile was allowed to enter the laboratory and deal with the samples and its products.

### DNA extraction

Teeth were incubated individually in 2% (pure solution) DanKlorix (Colgate-Palmolive) for decontamination of the surface and ground into powder using the MixerMill MM200 (Retsch, Haan, Gemany). Prior to DNA extraction, decalcification was performed. Bone powder (0.1 mg per sample) was mixed with 500 µL EDTA (0.5 M, pH = 8.0) and incubated at 37°C for 18 hours in a thermomixer. Then, 20 µL proteinase K (20mg/mL) were added and samples were incubated at 56°C as described (28). To separate the non-solved bone powder (if present) from the remaining liquid samples were centrifuged for 3-minute at 6000 upm. 200 µL of the resulting supernatant were subjected to an automated extraction step using the DNA Tissue Kit from the BioRobot EZ1 system (Qiagen, Hilden, Germany) following the manufacturer´s trace protocol. DNA was eluted in 50 µL 1 × TE buffer. The samples were immediately subjected to PCR or stored at -20°C. Negative extraction controls were always included in every run containing all reagents necessary for preparation and passing through all preparation steps.

### STR profiling

STR typing was done using the PowerPlex 16, 16HS, ES, ESI17, ESX17, S5 (all Promega), AmpFiSTR Identifiler, NGM, and SEfiler (all Applied Biosystems, Darmstadt, Germany). All kits were employed according to the manufacturer’s protocol with increased cycle numbers starting from 35 on a mastercycler gradient PCR machine (Eppendorf, Hamburg, Germany). From every sample, 5 µL of the DNA extract was subjected to the PCR as a template without prior quantification. Capillary electrophoresis was performed on an ABI Prism 3130 Genetic Analyzer (Applied Biosystems) with the following components: 11.7 µL HiDi-Formamid (Applied Biosystems), 0.3 µL size-standard (Applied Biosystems or Promega), and 0.5 µL PCR-product per sample. Detailed information on fragment analysis and assays used can be found in the supplementary table (supplementary table 1). Data were collected using the ABI Prism Genemapper Software Version 3.2 (Applied Biosystems) with a defined peak amplification threshold of 50 relative fluorescent units (rfu).

### Data interpretation and evaluation

For evaluation and comparison from all PCR kits the average of detected STR systems was calculated in percent (eg, 5 out of 15 = 33%, 1 out of 4 = 25%). For each kit, all detected systems were counted, independently of hetero- or homozygosity. Thereby, the peak high had to be at least 50 rfu, with a clear and smooth background.

Additionally, the success rate of correctly typed heterozygous alleles was determined. Signals were regarded as truly heterozygous when both alleles were detected independently at least twice. These correctly typed systems were counted and the percent success rate was calculated taking into account the number of markers for each kit.

We also analyzed the sex-determining marker amelogenin, which is included in every tested PCR kit. A reproducible detection of the X-specific fragment (X/-), only the Y-specific fragment (-/Y), and the full male profile (X/Y) was regarded as successful sex determination. Evaluation and comparison of this success rate were done in the same way as described before for the heterozygous detection results.

## Results

**Figure 1:**
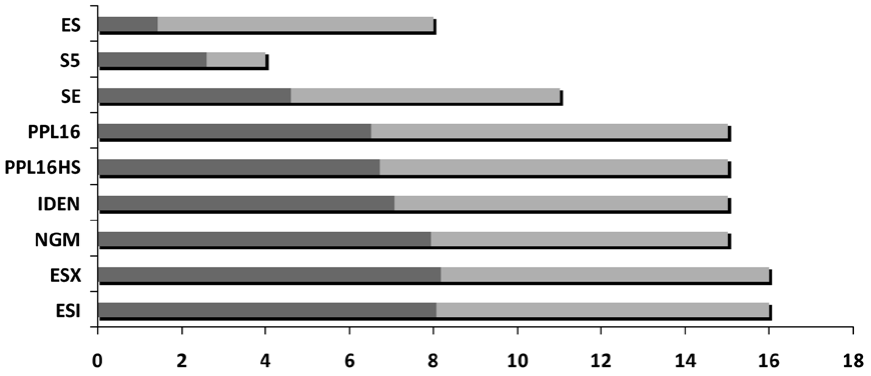
Average detection rate of each short tandem repeat (STR) kit. The results of detection of specific alleles with 9 different multiplex kits. Illustrated are the total STR markers of each kit (gray) and the average of detected STR markers (dark gray). ESI – PowerPlex ESI17, ESX – PowerPlex ESX17, PPL16/HS – PowerPlex 16/ 16HS, S5 – PowerPlex S5, ES – PowerPlex ES, NGM – AmpFiSTR NGM, SE – AmpFiSTR SEfiler, ID – AmpFiSTR Identifiler.

The greatest overall amplification success was obtained with the ESI, ESX (Promega), and NGM (Applied Biosystems) kit, with an average of approximately 8 STR detectable markers. Considering the total number of STR systems provided in each kit, the S5 kit (52%) yielded a comparable number of successfully typed markers to the ESI, ESX, and NGM kits (50%-53%) (Figure 1).

Predominantly detected were STR markers with small amplicon sizes (50-200 bp). This emphasizes the advantageous application of mini STRs when dealing with highly degraded material. A connection between the specific labeling of the amplicons and the success rate of the STR markers was not found.

**Figure 2:**
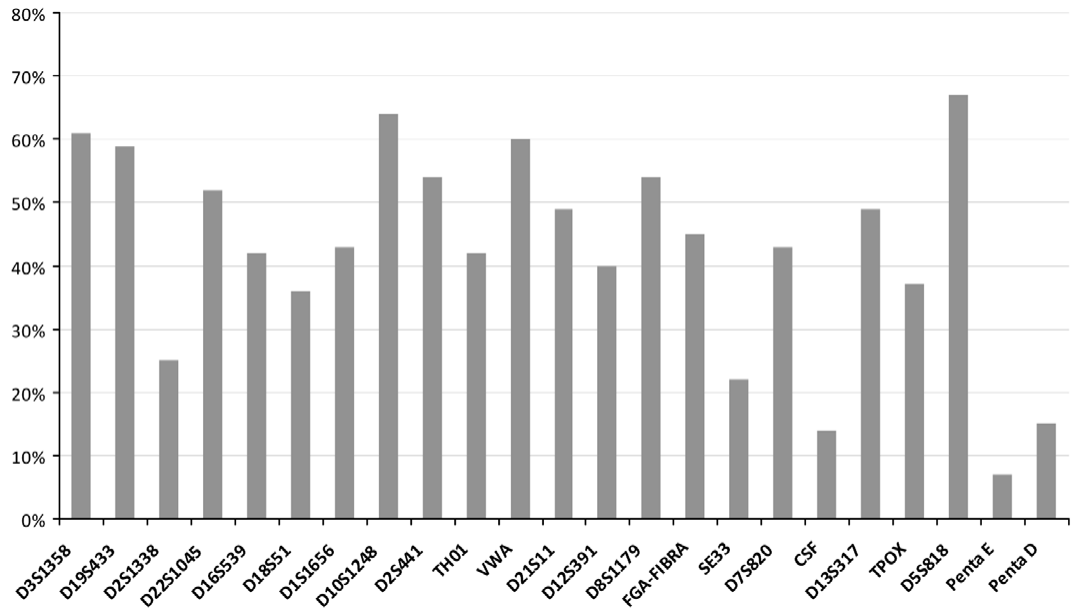
Average success rate of each short tandem repeat (STR) marker. The percentages of reproducibly detected markers for every locus. ESI – PowerPlex ESI17, ESX – PowerPlex ESX17, PPL16/HS – PowerPlex 16/ 16HS, S5 – PowerPlex S5, ES – PowerPlex ES, NGM – AmpFiSTR NGM, SE – AmpFiSTR SEfiler, ID – AmpFiSTR Identifiler.

When detection rate of each STR-marker was analyzed, the markers D3S1358, D10S1248, and D5S818 achieved the highest rates with over 60% (Figure 2). With exception of D10S1248 in the ESI Kit, the amplicons of these three markers were smaller than 200 bp (D3S1358 ≈ 90-150 bp, D10S1248 ≈ 50-120 bp (ESI = 275-330 bp), D5S818 ≈ 115-190 bp), which probably explains the good typing results.

ESI, ESX, and NGM kit showed similar typing success rates and informative values. Therefore, a more detailed analysis of the results was conducted regarding the occurrence of allelic drop-outs and drop-ins. As mentioned above, we decided on “truly heterozygous” when both alleles were detected in at least two different PCR runs, which enabled obtaining as authentic as possible profile. When counting those defined heterozygous markers, the ESI, ESX, and NGM kit showed the best results with 38%, 37%, and 36%, respectively (Table 1). The other assays were less reproducible with only 18%-30%. Regarding this, the data showed many allelic drop-outs continuously visible in all tested STR-kits. There was no significant difference between the 9 assays.

**Table 1:** Exemplarily short tandem repeat (STR)-typing results and final interpretation from one ancient DNA sample using three different polymerase chain reaction multiplex kits. For the evaluation of correctly typed heterozygous alleles, the results were interpreted as follows: bold – correctly and completely typed, regular –incompletely typed. ESI – PowerPlex ESI17; ESX – PowerPlex ESX17; NGM –AmpFiSTR NGM.

STR-kit	Sample	STR marker																
		D3S1358	D19S433	D2S1338	D22S1045	D16S539	D18S51	D1S1656	D10S1248	D2S441	TH01	VWA	D21S11	D12S391	D8S1179	FGA	SE33	Amelogenin
**ESI**	**extract a**	/	/	/	/	11/-	/	/	/	/	9.3/-	/	/	/	**10/14**	22	/	**X/Y**
	**extract b**	15/-	16/-	/	16/-	/	16/-	15/-	/	/	9.3/-	/	30/-	/	/	**21/22**	/	/
**NGM**	**extract a**	15/-	16/-	/	16/-	/	16/-	/	14/-	11/-	/	18/-	/	/	/	/	/	**X/Y**
	**extract b**	/	14/16	/	16/-	/	16/-	/	14/-	**11/14**	9.3/-	/	30/-	21/-	**10/14**	21	/	X
**ESX**	**extract a**	/	16/-	/	16/-	11/-	/	15/-	14/-	11/-	/	**18/19**	/	/	/	/	/	X
	**extract b**	15/-	16/-	21/-	16/-	/	/	15/-	14/-	**11/14**	/	**18/19**	/	/	/	21	/	Y
**Combined**	**final statement**	15/-	16/-	/	16/-	11/-	16/-	15/-	14/-	11/14	9.3/-	18/19	30/-	/	10/14	21/22	/	X/Y

**Figure 3:**
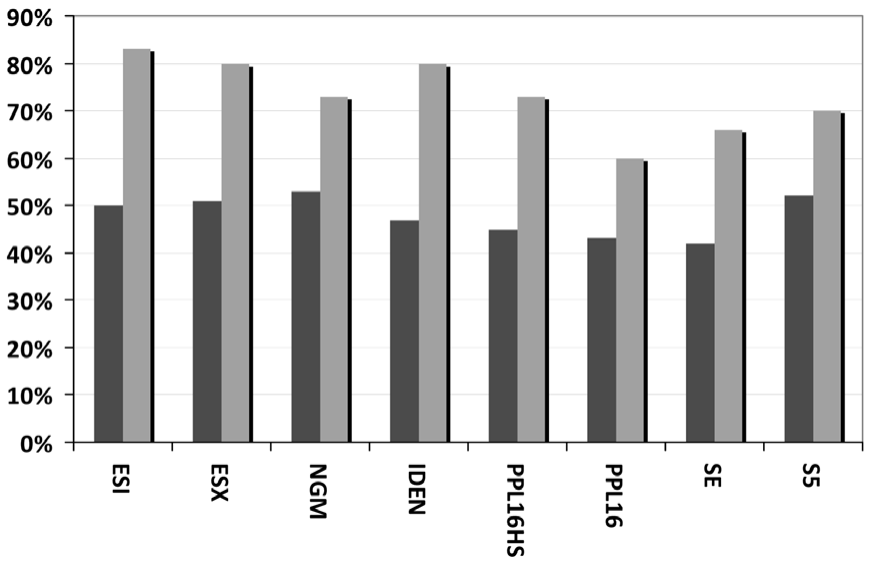
Average of reproducibly detected heterozygous (dark gray) and amelogenin (gray) alleles in percent. ESI – PowerPlex ESI17, ESX – PowerPlex ESX17, PPL16/HS – PowerPlex 16/ 16HS, S5 – PowerPlex S5, ES – PowerPlex ES, NGM – AmpFiSTR NGM, SE – AmpFiSTR SEfiler, ID – AmpFiSTR Identifiler.

Because of its high importance, the sex marker amelogenin was additionally thoroughly evaluated. The ESI kit showed the highest sex determination success rate of 83%, the ESX and the Identifiler kit of 80%, and the NGM and Powerplex 16 kit of 73%. The other tested kits only achieved detection rates between 40 and 66% (Figure 3).

## Discussion

Our study showed that the ESI, ESX, and NGM kits showed the best overall results regarding amplification success, detection rate, identification of heterozygous alleles, sex determination, and reproducibility of the obtained data.

When working with highly degraded DNA, several problems can occur and were therefore considered in our study. One important quality feature was the occurrence of drop-outs, which were more frequent in larger STR-amplicons. This is often described as a common phenomenon in highly degraded DNA samples from crime scenes or archeological findings (29,30). Next to allelic drop-outs, drop-ins also present a well-known problem. However, when encountering an unknown profile drop-ins can usually not be reliably identified and therefore no precise statement for the occurrence of allelic drop-ins can be made regarding the analyzed individuals. Thereby, the detection of real heterozygous alleles is a challenge, in most cases involving highly degraded DNA material due to allelic drop ins, -outs, and stutter artifacts (31).

When comparing the overall detection rate of different STR kits, the total number of STRs has to be kept in mind. The relatively high amplification rate of 50% for the S5 kit for example was less significant and meaningful than that of the other STR kits with a considerably greater number of STR markers. Therefore, the S5 kit is more useful and recommendable in terms of a screening PCR when the presence of DNA has to be checked and no other self-made screening PCR, which of course would be less expensive, is available (32).

ESX and NGM kit have displayed a high detection rate not only in our study – they have recently shown good results in the analysis of bone materials from World War II skeletal remains (33). This shows that the newly established kits are especially useful for an ancient DNA typing and not only for the forensic use.

The additional evaluation of the detection rate of heterozygous and sex determination alleles supports also the applicability of the ESI, ESX, and NGM kit for the analysis of ancient and highly degraded material. Taking into account the time and costs, ESI, ESX, and NGM kit differed only a little.

Our results emphasize the difficulty of STR typing from highly degraded ancient material and stress the necessity for a thorough and strict data evaluation. After comparing a high number of commercially available PCR multiplex kits, we feel certain to recommend the employment of at least two different kits, preferably from different companies or at least with different primer sequences to avoid false homozygous patterns due to primer binding mutations (34). Additionally, every analysis should be done at least in duplets, preferably triplets. NGM, ESI, and ESX kit were reliable assays and suited for the analysis of aDNA. With the exception of SE33, which was not part of the NGM kit, all assays contained identical STR-markers, required for the European DNA databases, using different primer-sets. In order to reduce or exclude null alleles and to increase the detection rate of highly degraded DNA, the simultaneous application of different STR multiplex kits is highly recommended (34-36). Due to the missing SE33 marker in the NGM assay and the similarity between the amplicon sizes of equal markers in the ESX and NGM kit, the combinations ESI + ESX or ESI + NGM kit should be preferred for the analysis of highly degraded ancient material.
