# Supplementary material for: STR-typing of ancient skeletal remains: which multiplex-PCR kit is the best?
Source: Croat Med J. 2012 Oct;53(5):416–22. doi: 10.3325/cmj.2012.53.416 (PMC3494155; doi:10.3325/cmj.2012.53.416)
Supplement: Supplementary Table 1 [file CroatMedJ_53_s012.pdf]

	Promega (Injection time ABI 3130: 3 kW, 3 sec)					Applied Biosystems (Injection time ABI 3130: 3 kW, 3 sec)		
		ESX	PPL16/HS	ES	S5	SE	ID	NGM
Amelogenin	Fluorescein 80-90	Fluorescein 80-90	TMR-ET 100-110	TMR-ET 100-110	Fluorescein 80-110	VIC 100-110	PET 100-110	VIC 100-110
D3S1358	Fluorescein 90-150	Fluorescein 90-150	Fluorescein 99-147	Fluorescein 100-150	/	FAM 90-140	VIC 97-145	PET 115-165
D19S433	Fluorescein 150-220	CXR-ET 190-260	/	/	/	NED 90-140	NED 92-150	NED 120-160
D2S1338	Fluorescein 220-295	JOE 180-260	/	/	/	FAM 277-345	VIC 291-359	FAM 280-360
D22S1045	Fluorescein 295-345	TMR-ET 50-120	/	/	/	/	/	NED 80-120
D16S539	JOE 50-130	JOE 260-330	JOE 260-308	/	/	FAM 220-270	VIC 250-296	FAM 220-280
D18S51	JOE 130-210	Fluorescein 270-370	Fluorescein 280-360	Fluorescein 270-370	Fluorescein 110-200	PET 265-335	NED 264-351	VIC 260-350
D1S1656	JOE 210-275	JOE 125-180	/		/	/	/	PET 170-220
D10S1248	JOE 275-330	JOE 50-125	/	/	/	/	/	FAM 70-120
D2S441	JOE 330-380	CXR-ET 60-125	/	/	/	/	/	PET 80-115
TH01	TMR-ET 40-125	Fluorescein 150-195	Fluorescein 152-196	Fluorescein 150-195	JOE 80-135	NED 155-200	VIC 160-210	NED 170-220
VWA	TMR-ET 120-195	TMR-ET 120-200	TMR-ET 123-183	TMR-ET 120-180	/	FAM 140-210	NED 152-210	FAM 150-200
D21S11	TMR-ET 195-270	Fluorescein 195-270	Fluorescein 155-273	Fluorescein 195-260	/	PET 180-250	FAM 175-256	VIC 180-250
D12S391	TMR-ET 270-350	CXR-ET 125-190	/	/	/	/	/	PET 220-290
D8S1179	CXR-ET 50-140	TMR-ET 200-260	TMR-ET 200-250	TMR-ET 200-250	Fluorescein 200-300	VIC 124-170	FAM 123-175	VIC 120-180
FGA	CXR-ET 140-300	TMR-ET 260-420	TMR-ET 308-464	TMR-ET 305-465	JOE 135-300	NED 200-360	PET 196-352	NED 230-360
SE33	CXR-ET 300-500	CXR-ET 260-460	/	JOE 195-330	/	VIC 190-350	/	/
D7S820	/	/	JOE 211-260	/	/	/	FAM 256-300	/
CSF	/	/	JOE 317-361	/	/	/	FAM 300-350	/
D13S317	/	/	JOE 160-205	/	/	/	VIC 210-250	/
TPOX	/	/	TMR-ET 254-302	/	/	/	NED 210-250	/
D5S818	/	/	JOE 115-160	/	/	/	PET 130-190	/
Penta E	/	/	Fluorescein 379-484	/	/	/	/	/
Penta D	/	/	JOE 370-454	/	/	/	/	/
